# Jaw Periosteal Cells Seeded in Beta-Tricalcium Phosphate Inhibit Dendritic Cell Maturation

**DOI:** 10.3390/biom10060887

**Published:** 2020-06-10

**Authors:** Jingtao Dai, Felix Umrath, Siegmar Reinert, Dorothea Alexander

**Affiliations:** Department of Oral and Maxillofacial Surgery, University Hospital Tübingen, Osianderstr 2-8, 72076 Tübingen, Germany; jingtao.dai@med.uni-tuebingen.de (J.D.); felix.umrath@med.uni-tuebingen.de (F.U.); siegmar.reinert@med.uni-tuebingen.de (S.R.)

**Keywords:** jaw periosteal cells, beta-tricalcium phosphate, dendritic cell maturation, osteogenesis

## Abstract

Mesenchymal stem cells (MSCs) have gained attraction not only in the field of regenerative medicine but also in the field of autoimmune disease therapies or organ transplantation due to their immunoregulatory and/or immunosuppressive features. Dendritic cells (DCs) play a crucial role in initiating and regulating immune reactions by promoting antigen-specific T cell activation. In this study, we investigated the effect of human jaw periosteal progenitor cells (JPCs) seeded in beta-tricalcium phosphate (β-TCP) scaffolds on monocyte-derived DC differentiation. Significantly lower numbers of differentiated DCs were observed in the presence of normal (Co) and osteogenically induced (Ob) JPCs-seeded β-TCP constructs. Gene expression analysis revealed significantly lower interleukin-12 subunit p35 (IL-12p35) and interleukin-12 receptor beta 2 (IL-12Rβ2) and pro-inflammatory cytokine interferon-gamma (IFN-γ) levels in DCs under Ob conditions, while interleukin-8 (IL-8) gene levels were significantly increased. Furthermore, in the presence of JPCs-seeded β-TCP constructs, interleukin-10 (IL-10) gene expression was significantly induced in DCs, particularly under Ob conditions. Analysis of DC protein levels shows that granulocyte-colony stimulating factor (G-CSF) was significantly upregulated in coculture groups. Our results indicate that undifferentiated and osteogenically induced JPCs-seeded β-TCP constructs have an overall inhibitory effect on monocyte-derived DC maturation.

## 1. Introduction

Stem cell-based tissue engineering (TE) is a promising strategy for bone tissue regeneration, overcoming drawbacks associated with bone autografting such as complex surgical procedures, limited availability, and high costs [1,2]. In bone TE, MSCs isolated from bone marrow and other tissues not only possess the ability of multipotent differentiation capacity but also promote tissue homeostasis, metabolism, growth, and repair. Notably, periosteum-derived cells can be a source of stem cells with great potential for bone and cartilage repair [3,4,5,6,7]. Compared to other skeletal stem cells, human jaw periosteal cells (JPCs) could represent the most suitable stem cell source in the field of oral bone regeneration. The advantages of JPCs are simple tissue harvesting and cell isolation. In our previous studies, we optimized cell culture conditions, biomaterials, and detection methods for the mineralization potential of JPCs in order to move further step by step towards bone regenerative clinical applications [8,9,10,11,12,13].

The scaffold type plays a critical role in bone TE, possessing not only structural function for the colonializing cells but also providing mechanical stability, particularly in the masticatory region. β-TCP has been widely used in clinical applications because of its excellent biocompatibility and osteoconductive ability [14,15,16,17]. Additionally, β-TCP has moderate biodegradability in physiological environments. The β-TCP scaffold can be degraded by acidic products released by surrounding cells such as osteoclasts or macrophages, and substantially replaced by newly formed bone [14,15]. Previously, cocultures of MSCs and MSC-derived endothelial cells seeded in porous β-TCP scaffolds resulted in successful repair of large segmental bone defects in a rabbit model [16]. Regarding periosteal cells, Vacanti et al. firstly reported a successful clinical case using periosteal cells combined with hydrogel and a calcium phosphate scaffold for bone regeneration [17]. Our group previously demonstrated that JPCs seeded into biofunctionalized β-TCP scaffolds could represent a suitable strategy for the development of bone TE constructs [18,19].

DCs have been recognized as the most potent antigen-presenting cells (APCs) widely distributed in the human body [20]. DCs can be derived from human peripheral blood monocytes (PBMCs) through culturing with granulocyte-macrophage colony-stimulating factor (GM-CSF) and interleukin-4 (IL-4) [21,22,23]. DCs play a critical role in the initiation of primary immune responses and tolerance induction [24]. In fact, DCs can be divided into two major types: an immature and a mature stage, respectively [24,25] The mature stage of DCs is related to a high expression of costimulatory molecules involved in antigen presentation (i.e., CD80, CD86, and MHC-II). Undoubtedly, DC maturation is a fundamental prerequisite for the induction of immunogenic T cell responses [20]. Numerous studies have proven that MSCs not only can inhibit DC maturation [26,27] but also can inhibit T cells proliferation [28,29] due to their low immunogenicity and immunoregulatory properties. Previously, we reported that JPCs exhibit an overall inhibiting effect on DC maturation in a 2D Transwell system [30].

In the present study, we investigated the effects of JPCs seeded within β-TCP constructs on human DC maturation. Therefore, gene expression, phenotype, and differentiation of DCs were investigated under cocultivation with untreated or osteogenically induced JPCs cultured within β-TCP constructs.

## 2. Materials and Methods

### 2.1. Isolation of JPCs and PBMCs

Samples from three donors were included in the study, with the approval number 194/2008BO2 from the local ethics committee. After obtaining written informed consent from these three donors, human JPCs were isolated and cultured, as mentioned previously [18,30]. JPCs were cultured in Dulbecco’s Modified Eagle Medium/Nutrient Mixture F-12 (DMEM/F-12, Invitrogen/Thermo Fisher Scientific, Waltham, USA) containing 10% fetal calf serum (FCS) (Sigma-Aldrich, Darmstadt, Germany), 1% penicillin/streptomycin (Lonza, Basel, Switzerland), and 1% fungicides (Biochrom AG, Berlin, Germany) under standard cell culture conditions. Pooled JPCs of these three donors from the sixth passage were used for all coculture experiments, and cell culture medium was replaced every other day. The osteogenic differentiation potential of the used JPCs was evaluated as reported before [30].

The human peripheral blood was collected and diluted with Dulbecco’s phosphate-buffered saline (DPBS, Sigma-Aldrich, Darmstadt, Germany), and layered over 12 mL of 1.077 g/mL Ficoll-Paque PLUS (GE Healthcare Europe GmbH, Freiburg, Germany). Plasma and red blood cells were separated from PBMCs by density gradient centrifugation (no break, 810× *g*, 20 °C) for 20 min. PBMCs were harvested and pipetted into another fresh 50 mL tube. The cells were washed 3 times with DPBS, and then a cell density of 1 × 10^6^ cells/well was cultivated in x-vivo serum-free medium (Biozym, Hessisch-Oldendorf, Germany) with 3% autologous plasma and 1% penicillin/streptomycin for 24 h.

### 2.2. Cell Seeding of β-TCP Constructs

JPCs in culture flasks were trypsinized (Trypsin-Versene EDTA, Lonza, Basel, Switzerland) and pooled JPCs of these three donors were seeded in β-TCP constructs (Curasan AG, Kleinostheim, Germany). The constructs were preincubated in 96-well polypropylene culture plates containing DMEM/F-12 media for 1 h. A starting density of 1 × 10^5^ cells/scaffold was seeded into β-TCP constructs in 50 μL volume; the JPCs-seeded β-TCP scaffolds were incubated for 2 h at 37°C. After 2 h of incubation, additional 150 μL medium were subsequently pipetted to overlay JPCs-seeded β-TCP constructs.

For osteogenic differentiation of JPCs-seeded constructs before coculture experiments, the constructs were cultured in a 96-well plate under osteogenic (Ob) conditions (DMEM/F-12 complete medium containing 4 μM dexamethasone, 100 μM L-ascorbic acid 2-phosphate, and 10 mM β-glycerophosphate, Sigma-Aldrich, Germany) for 7 days. JPCs-seeded β-TCP scaffolds cultured with Co (untreated) medium for the same time period served as undifferentiated controls. The medium was replaced every 2 days. The osteogenesis-relevant genes by JPCs cultured within β-TCP constructs and cocultured with DCs were quantified. Supplemental Figure S1 shows gene expression levels of ALP and RUNX2 after 14 days (7 days monoculture and 7 days coculture) of osteogenic differentiation.

### 2.3. Coculture of JPCs-Seeded Constructs and Monocytes

For cocultivation experiments, 6-well Transwell coculture plates (0.4 μm pore size membrane, Corning, Wiesbaden, Germany) were chosen. PBMCs were incubated for 24 h after cell seeding in the lower compartment, and medium change was performed using autologous plasma-x-vivo medium containing 40 ng/mL IL-4 and 100 ng/mL GM-CSF (Sigma-Aldrich, Darmstadt, Germany) in order to begin DC differentiation. Coculture experiments were performed in the absence or presence of JPCs-seeded or cell-free constructs. Therefore, four scaffolds were placed per upper compartment under undifferentiated or osteogenic media. Four different groups were included into the upper compartment: 1. JPCs-free constructs cultured under undifferentiated DMEM/F-12 media conditions (β-TCP/Co), 2. JPCs-free constructs cultured under osteogenic medium (β-TCP/Ob), 3. JPCs-seeded constructs cultured under undifferentiated DMEM/F-12 media conditions (JPCs + β-TCP/Co), and 4. JPCs-seeded constructs cultured under osteogenic conditions (JPCs + β-TCP/Ob). At day 3, media change in the upper compartment was performed. At day 6, the second DC differentiation cocktail containing 40 ng/mL IL-4, 100 ng/mL GM-CSF, 10 ng/mL TNF-α, 10 ng/mL IL-1β, 10 ng/mL IL-6 (Tebu Bio, Offenbach, Germany), and 1 μg/mL PGE2 (Bio Trend, Köln, Germany) was added to the x-vivo medium containing autologous plasma for another 24 h to stimulate DCs maturation.

### 2.4. Analysis of Cell Viability Using Propidium Iodide

After 7 days of coculture, the suspension (mature DCs) and adherent (monocytes/immature DCs) cells of the lower compartment were harvested into separated 50 mL tubes and centrifugated for 7 min (1400×*g*, 8 °C). The cell pellet was resuspended in 1 mL DMEM/F-12 medium, and 100 μL of cell suspension was added into a FACS tube containing 100 μL DPBS. Then, 1 μL of 1 mg/mL propidium iodide (PI) staining solution was added to the sample, mixed gently, and incubated on ice in dark for 1 min. Following this method, the suspension and adherent cells were counted and analyzed by flow cytometry, respectively. The ratios of suspension/adherent cells under different culture conditions were calculated.

### 2.5. Flow Cytometric Analyses of Dendritic Marker Expression

Monocytes before coculture (day 1) and DCs after coculture (day 7) were collected for flow cytometric analysis and served as controls. After centrifugation for 7 min (1400× *g*, 8 °C), cell pellets were resuspended in 10% Gamunex (human immune globulin solution, Talecris Biotherapeutics, Germany), and incubated for 15 min on ice. After this incubation period time, the cell density was adjusted to 2 × 10^6^ cells/mL with FACS buffer (DPBS, 0.1% BSA, and 0.1% sodium azide). Cells were incubated on ice with the specific phycoerythrin (PE)-labeled mouse antihuman HLA-DR (MACS Miltenyi Biotec, Bergisch Gladbach, Germany), and allophycocyanin (APC)-labeled mouse antihuman CD14, CD83, and CD209 (BioLegend, San Diego, USA) antibodies for 15 min in dark. Subsequently, labeled cells were washed two times with FACS buffer and centrifuged for 7 min (1400 rpm, 8 °C). Cell pellets were resuspended in 200 μL FACS buffer and analyzed by flow cytometry using the Guava easyCyte 6HT-2L Instrument (Merck Millipore, Darmstadt, Germany). For data evaluation, the guavaSoft 2.2.3 (InCyte 2.2.2, Luminex Corporation, Chicago, IL, USA software was used.

### 2.6. RNA Isolation and Quantitative Gene Expression Analyses in DCs

RNA was isolated from DCs (after 7 days of differentiation) using the NucleoSpin RNA XS kit (Macherey-Nagel, Hoerd, France) as recommended by the manufacturer and photometrically measured and quantified using the Qubit RNA Broad-Range (BR) Assay Kit and a corresponding 3.0 fluorometer (Invitrogen/Thermo Fisher Scientific, Waltham, USA). Subsequently, cDNA synthesis was performed using 200 ng of RNA and the SuperScript VILO Kit (Invitrogen/Thermo Fisher Scientific, Waltham, USA), as recommended by the manufacturer. To quantify mRNA expression levels, the real-time LightCycler System (Roche Diagnostics, Mannheim, Germany) was used. For the PCR reactions, DEPC-treated water, DNA Master SYBR Green I kit (Roche, Mannheim, Germany), and commercial primer kits (IL-12p35, IL-12p40, IL-12Rβ1, IL-12Rβ2, IFN-γ, TNF-α, IL-4, IL-8, IL-10, MIP-1α, MIP-1β, and G-CSF) from Search LC (Heidelberg, Germany) were used for 40 amplification cycles of the target DNA. The target gene transcript levels were normalized to those of the housekeeping gene GAPDH (Search LC, Heidelberg, Germany). Finally, the ratio of the β-TCP/Co group was set as 1 (control), and x-fold induction indices in relation to this control were calculated.

### 2.7. Proteome Profiler Array

To measure the expression of different proteins in DCs (lower chamber) or JPCs (upper chamber) supernatants at day 1 and 7 of DC differentiation and G-CSF levels in supernatants from JPCs (cultivation in the absence of DCs) cultured with human platelet lysate (hPL) under Co/Ob conditions (with or without dexamethasone) at day 15, proteome profiler array kits (Human Cytokine Array Kit, Human Soluble Receptor Array Kit, and Non-Hematopoietic Panel; R&D Systems, Germany) were used, following the manufacturer’s instructions. Briefly, the membranes were blocked with respective array buffer for 1 h at room temperature (RT) and then incubated with 1.5 mL of sample/array buffer/detection antibody mixtures overnight at 4 °C. After washing three times, the membranes were incubated with 2 mL of diluted streptavidin–HRP at RT for 30 min. After three washing steps again, the membranes were exposed to 1 mL of the chemiluminescent reagent mixture and the radiographic films for 10 min. The developed films were scanned, and data analysis of positive signals was carried out using the ImageJ software.

### 2.8. Statistical Analysis

The statistical values for all measurements are expressed as means ± standard error of means (SEM). Student’s t-tests or one-way analysis of variance (ANOVA) for repeated measurements followed by Tukey’s multiple comparisons tests were used. All statistical analyses were carried out using the SPSS V22.0 software (IBM Corp., New York, NY, USA). A value of *p* < 0.05 (two-tailed) was considered as statistically significant.

## 3. Results

### 3.1. Cell Morphology of DCs

**Figure 1:**
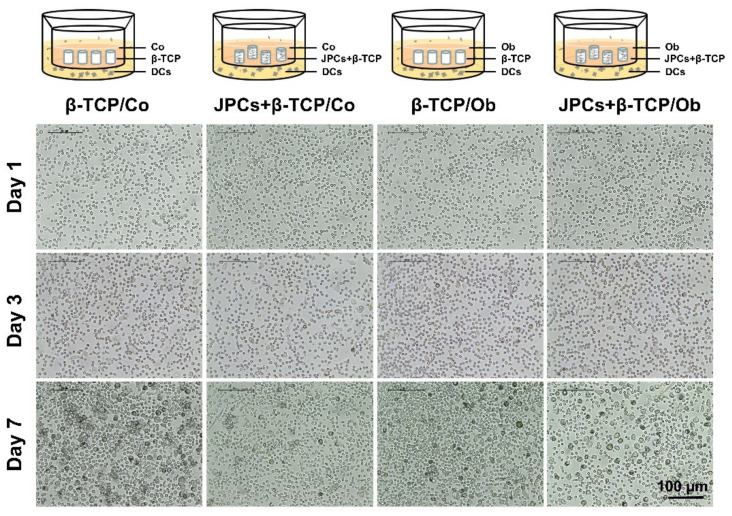
Representative microscopic images showing the morphologic characteristics of monocytes and dendritic cells (DCs) in unseeded (beta-tricalcium phosphate, β-TCP) and jaw periosteal progenitor cells (JPC)-seeded constructs (JPCs + β-TCP) under untreated (Co) or osteogenic (Ob) conditions at day 1, 3, and 7 of DC differentiation, respectively.

To investigate the effects of JPCs-seeded β-TCP constructs on DC maturation, cell-seeded scaffolds were cultured under untreated (Co) and osteogenic conditions (Ob) for 14 days. Representative microscopic images indicated the morphologic appearance of monocytes and DCs. The monocytes were observed as small round cells among all groups at day 1, whereas differentiating monocytes were found to be getting bigger at day 7, as shown in Figure 1. No obvious larger cell morphologies were observed in the presence of JPCs-seeded β-TCP constructs under both culture conditions, implying relatively less differentiated DCs.

### 3.2. DC Densities in Mono- (β-TCP/Co and β-TCP/Ob) and Cocultures (JPCs + β-TCP/Co and Ob)

**Figure 2:**
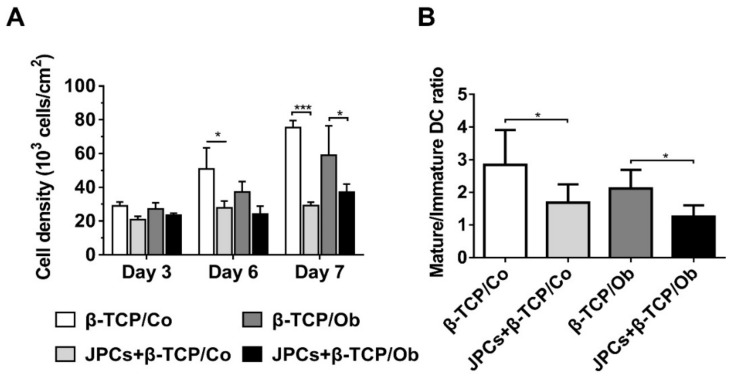
DC densities and mature/immature DC ratios in monocultures (β-TCP-groups) and cocultures (JPCs + β-TCP) preincubated under untreated (Co) and osteogenic (Ob) conditions for 7 days. (**A**) DC densities (10^3^ cells/cm^2^) were quantified using ImageJ software. (**B**) The ratio of suspension (mature)/adherent (immature) cells in the lower chamber was determined by flow cytometry using propidium iodide (PI) staining after DC differentiation at day 7. Data were collected from five independent experiments (* *p <* 0.05, *** *p* < 0.001).

DC densities (adherent cells within the lower compartment) and the ratio of suspension/adherent cells were investigated in mono- (cocultivation with cell-free scaffolds) and cocultures (with JPCs-seeded β-TCP constructs). Compared to monocultures (β-TCP/Co), DC densities were significantly lower in untreated cocultures (JPCs + β-TCP/Co) at day 6 (JPCs + β-TCP/Co 27.76 ± 2.40 versus β-TCP/Co 50.90 ± 7.26, *p <* 0.05), as shown in Figure 2A. Similarly, at day 7, DC densities in cocultures (JPCs + β-TCP) were shown to be significantly lower than the counterparts of monocultures under both conditions (JPCs + β-TCP/Co 29.19 ± 1.19 versus β-TCP/Co 75.34 ± 2.47, *p <* 0.05 and JPCs + β-TCP/Ob 37.09 ± 2.76 versus β-TCP/Ob 59.07 ± 10.06, *p <* 0.05, respectively). As illustrated in Figure 2B, compared to the β-TCP monoculture groups, the significantly lower ratios of suspension to adherent DCs were detected in the coculture (JPCs + β-TCP) groups under both culture conditions (Co/Ob). (JPCs + β-TCP/Co 1.69 ± 0.56 versus β-TCP/Co 2. 84 ± 1.07, *p <* 0.05 and JPCs + β-TCP/Ob 1.26 ± 0.34 versus β-TCP/Ob 2.12 ± 0.57, *p <* 0.05, respectively). 

### 3.3. Flow Cytometric DC Characterization 

**Figure 3:**
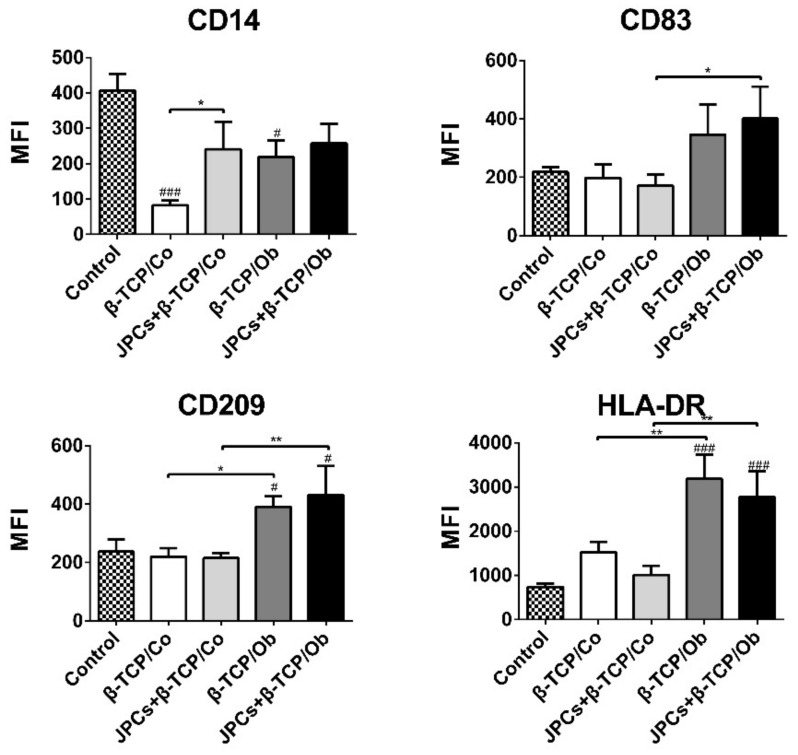
Flow cytometric analysis of DC surface marker expression in the undifferentiated state (monocyte control) and after 7 days DC differentiation in monocultures (β-TCP) and cocultures (JPCs + β-TCP) preincubated under Co and Ob conditions for initial 7 days (constructs were cultivated for 14 days in total). The mean fluorescence intensity (MFI) of positive cells is shown for the indicated markers CD14, CD83, CD209, and HLA-DR. Data were collected from five independent experiments (* *p <* 0.05, ** *p <* 0.01 # and ### represent *p <* 0.05 and *p <* 0.001 when compared to the undifferentiated monocyte control, respectively).

To compare the monocyte phenotype before and after cocultures, surface marker expression was detected in five independent experiments. Figure 3 shows significantly down-regulated expression (MFI) of CD14 on DCs in β-TCP (Co/Ob) groups compared to undifferentiated monocyte control. More importantly, CD14 protein expression was found to be significantly lower in monocultures (β-TCP) compared with cocultures with JPCs-seeded β-TCP constructs under normal conditions. In addition, MFIs for CD83, CD209, and HLA-DR expressions on differentiated DCs were significantly increased in monocultures (β-TCP-group) and cocultures (JPCs + β-TCP) under Ob compared to Co conditions. Similarly, MFIs of CD209 and HLA-DR were significantly upregulated in monocultures (β-TCP-group) and cocultures (JPCs + β-TCP) under Ob conditions compared to the undifferentiated monocyte control.

The percentages of positive cells for the four surface markers are listed in Table 1. DCs cocultured with cell-free β-TCP or JPCs-seeded β-TCP constructs under Ob conditions revealed significant higher percentages of CD14-positive cells compared to control and Co conditions. In addition, a similar trend could be found between the results of CD209 and CD14, whereas DCs cocultured with JPCs-seeded β-TCP constructs exhibited no statistically significant differences under Ob conditions in comparison to Co conditions. Additionally, HLA-DR was significantly upregulated in the groups cocultured with β-TCP and JPCs-seeded β-TCP constructs under both Co/Ob conditions compared to undifferentiated monocyte controls.

### 3.4. DC Gene Expression Analysis

**Figure 4:**
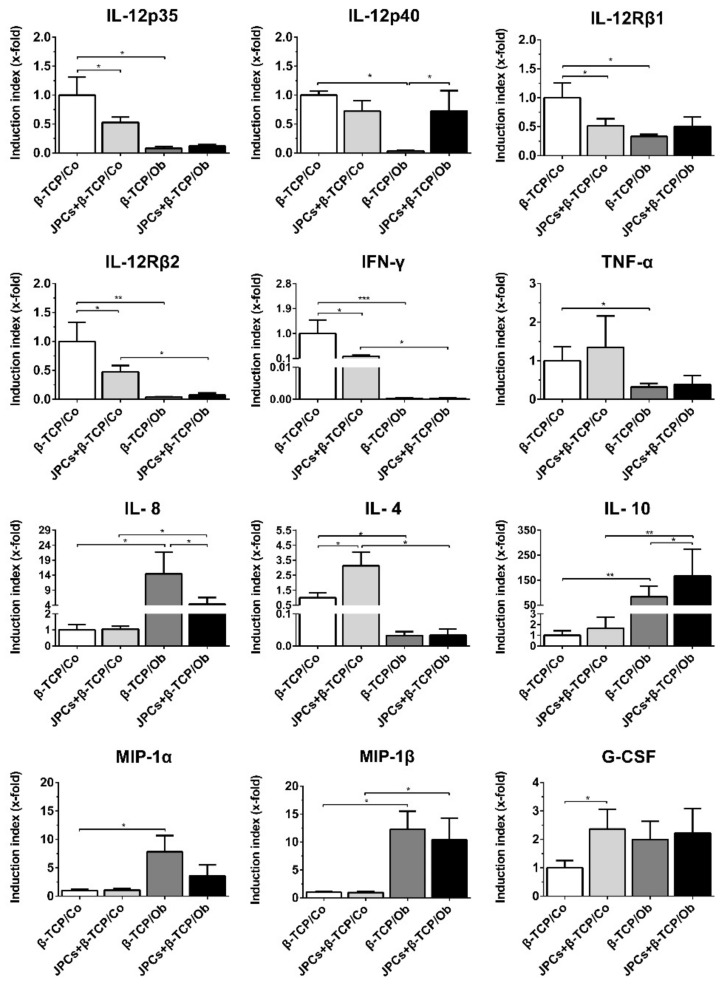
Quantitative gene expression of DCs cocultured with cell-free β-TCP and JPCs-seeded β-TCP constructs for 7 days after their preincubation under Co and Ob conditions for initial 7 days (constructs were cultivated for 14 days in total). Analyzed gene expressions were quantified by the LightCycler System, and ratios of genes of interest in relation to the housekeeping gene glyceraldehyde 3-phosphate dehydrogenase (GAPDH) were calculated. Gene levels in DCs cultured with cell-free β-TCP constructs under normal conditions (Co) was set as 1 (control), and induction indices (x-fold) in relation to the control were calculated. Data were collected from three independent experiments (* *p* < 0.05, ** *p* < 0.01, *** *p* < 0.001).

To evaluate the effect of cell-free or JPCs-seeded β-TCP constructs on DC gene expression, monocytes were differentiated in mono- and cocultures for 7 days after precultivation of JPCs under untreated and osteogenic conditions for 1 week. As shown in Figure 4, cocultured DCs (JPCs + β-TCP/Co) showed significant downregulation of four genes (IL-12p35, interleukin 12 receptor beta 1 (IL-12Rβ1), IL-12Rβ2, and IFN-γ) and upregulation of IL-4 and G-CSF compared with the expression of DCs cultured in monocultures (β-TCP/Co). In addition, DCs cultivated in coculture with osteogenically induced constructs (JPCs + β-TCP/Ob) showed significant downregulation of IL-8 and upregulation of interleukin-12 subunit p40 (IL-12p40) as well as of the anti-inflammatory IL-10 compared to DC expression in monocultures (β-TCP/Ob). Nevertheless, DCs exposed to cell-free β-TCP constructs under Ob conditions revealed a different gene expression pattern. Compared to DC expression in coculture with cell-free β-TCP constructs under untreated conditions, significant downregulation of the genes IL-12p35, IL-12p40, IL-12Rβ1, IL-12Rβ2, IFN-γ, TNF-α, and IL-4 and an upregulation of the genes IL-8, IL-10, macrophage inflammatory proteins-1α (MIP-1α), and macrophage inflammatory proteins-1β (MIP-1β) were detected, indicating that this gene regulation is only due to the effects of the osteogenic medium. Additionally, DCs cultured with JPCs + β-TCP constructs under Ob conditions showed a significantly downregulated gene expression of IL-12Rβ2, IFN-γ, and IL-4 and an upregulated gene expression of IL-8, IL-10, and MIP-1β compared to JPCs + β-TCP/Co.

In summary, when the effects of the osteogenic medium are deducted, JPCs seem to partially suppress IL-12p35, IL-12Rβ1, IL-12Rβ2, IFN-γ, and IL-8 gene expression, whereas JPCs as well as osteogenic medium seem to induce IL-4, IL-10, and G-CSF transcript levels.

### 3.5. DC Protein Expression Analysis

**Figure 5:**
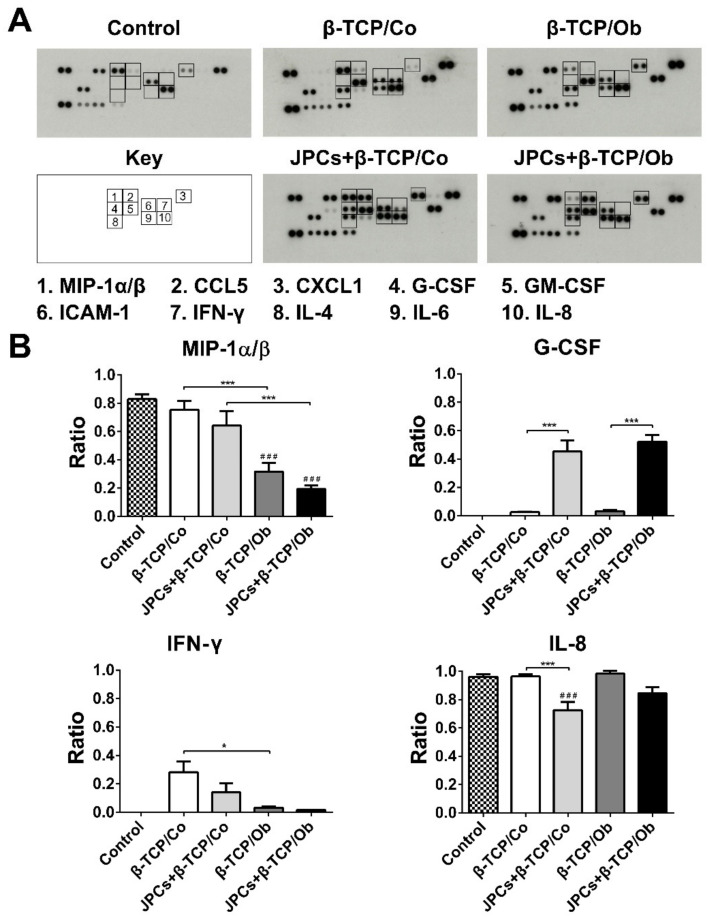
Protein expression of macrophage inflammatory proteins-1α/β (MIP-1α/β), interferon-gamma (IFN-γ), interleukin-8 (IL-8), and granulocyte-colony stimulating factor (G-CSF) in supernatants from peripheral blood monocytes (PBMCs) (control) and differentiated DCs (for 7 days) cocultured with cell-free β-TCP and JPCs-seeded β-TCP constructs after their precultivation under Co and Ob conditions for initial 7 days (constructs were cultivated for 14 days in total). (**A**) Representative images of proteome profiler arrays (Human Cytokine Array Kit) and (**B**) quantification analysis of the protein expression of the 4 marked proteins (MIP-1 α/β, G-CSF, IFN-γ, and IL-8) by ImageJ software. Data were collected from three independent experiments (* *p <* 0.05, *** *p <* 0.001; ### represents *p <* 0.001 when compared to the PBMCs control).

To identify the protein expression patterns in DCs, we used a human cytokine array comprising 36 human cytokines. Supernatants of DCs in coculture after 7 days of dendritic differentiation were collected and used for secretome analysis. The used cell-free and cell-seeded constructs were cultured under Co and Ob conditions for 7 days before cocultures with DCs were started and for additional 7 days in cocultures. As shown in Figure 5A, expression of MIP-1α/β and IFN-γ were decreased under Ob condition. Notably, the expression of G-CSF was increased in JPCs + β-TCP under both culture conditions. Figure 5B shows that the pro-inflammatory proteins MIP-1α/β and IFN-γ were significantly decreased in the β-TCP/Ob and JPCs + β-TCP/Ob groups compared to the corresponding Co groups, respectively. In the JPCs + β-TCP/Co group, the expression of IL-8 was significantly suppressed compared to the JPC-free β-TCP/Co group. Moreover, JPCs-seeded β-TCP constructs significantly induced the expression levels of G-CSF compared to cell-free β-TCP constructs under both culture conditions. Unfortunately, we were not able to detect IL12p70 levels. IL-4, IL-6, and GM-CSF were compounds of the DC maturation cocktail. Therefore, we performed no quantification of these cytokines.

## 4. Discussion

For bone regeneration, an ideal bone substitute should safely biodegrade in vivo and should be replaced by new bone tissue at the same time. It has been shown that β-TCP is a promising biomaterial, showing a balance between scaffold degradation and new bone formation in physiological environments [31,32]. Numerous studies have demonstrated that β-TCP possesses excellent biocompatibility and low immunogenicity [33,34]. Despite of the abovementioned advantages, the β-TCP material shows low osteoinductive abilities. Our previous studies reported that biofunctionalized β-TCP scaffolds improved functions of the colonializing cells [18,19]. Dendritic cells and macrophages are specialized antigen-presenting cells that initiate and orchestrate immune responses. In order to exclude rejection of our in vitro engineered constructs after implantation into the defect site, we decided to investigate the interactions between JPCs-seeded β-TCP scaffolds and DCs in the present study.

Dendritic cell maturation is mainly determined by stromal microenvironment [35,36]. Human bone marrow-derived MSCs possess the ability to inhibit human monocytic-derived DC differentiation [37]. In our previous study, we could demonstrate that undifferentiated and osteogenically differentiated two-dimensionally cultivated JPCs are potentially capable of suppressing DC maturation in the coculture [30]. In the present study, three-dimensionally cultured JPCs within β-TCP scaffolds significantly decreased DC numbers during DC maturation, as shown in Figure 1 and Figure 2A. Additionally, Figure 2B shows that the ratio of suspension cells (corresponding to mature DCs) to adherent cells (corresponding to immature DCs) was significantly lower when DCs were cocultured with JPCs-seeded β-TCP constructs compared to that of monocultures of the respective media. Therefore, we can assume that a higher number of monocytes might differentiate into DCs in monocultures (in the presence of β-TCP constructs) than in cocultures with JPCs-seeded constructs. For our experiments, we used the “standard” cytokine cocktail to induce final DC maturation. A more specific cocktail containing IL-1β, TNF-α, IFN-α, IFN-γ, and polyinosinic:polycytidylic acid can give rise to an α-type 1-polarized DC type expressing substantially higher levels of costimulatory molecules, as reported in several studies [38]. Since derived αDC1 cells display stronger immunostimulatory functions, we did not consider this aspect showing a limitation of our study.

Concerning the CD14 expression on differentiated DCs in the coculture with cell-free β-TCP scaffolds, we detected a significant decrease compared with the undifferentiated monocyte control, as shown in Figure 3. Notably, JPCs growing within β-TCP scaffolds could significantly upregulate CD14 expression compared to that in the presence of cell-free β-TCP constructs, under normal culture conditions, indicating that 3D-cultured JPCs inhibited monocytes differentiation into DCs. CD14 has been considered as a useful marker of monocytes. In addition, CD14+ monocytes in human peripheral blood play an important role in both innate and adaptive immunity. In that, CD14^+^ monocytes can recognize pathogens, do phagocytosis, and secrete cytokines, such as IL-1β, IL-10, and IL-6 [39]. Specifically, CD14^+^ monocytes can promote umbilical cord matrix stem cell-induced suppression of T cell proliferation probably through the IL-1β–PGE2 axis [40]. Our Figure 3 shows an increased expression of the markers CD83, CD209, and HLA-DR probably related to the osteogenic medium, especially by induction with the corticoid dexamethasone. Notably, a similar tendency was observed between the results of percentage of CD14-positive DCs (Table 1) and MFIs of HLA-DR expression (Figure 3). Hawrylowicz and coauthors reported in a previous work that dexamethasone can promote GM-CSF-induced HLA-DR antigen expression on monocytes [41]. In our experiment, DC differentiation cocktail contains IL-4, GM-CSF, and TNF-α, which can effectively upregulate CD83 of myeloid lineage cells as previously reported [42].

To further analyze the effects of JPCs, we investigated the gene expression of DCs cultured in the presence of β-TCP with or without cells. Figure 4 presents the overall inhibitory effects of JPCs-seeded β-TCP scaffolds on the gene expression of DCs. For the normal culture conditions, JPCs + β-TCP led to overall lower gene expression of IL-12p35, IL-12Rβ1, IL-12Rβ2, and the pro-inflammatory cytokine IFN-γ. We also detected significantly higher anti-inflammatory cytokine expression of IL-4 and G-CSF, whereas osteogenic conditions led to decreased pro-inflammatory IL-8 and increased anti-inflammatory IL-10 transcript levels. These data indicated that JPCs-seeded β-TCP scaffolds not only inhibit pro-inflammatory cytokine expression but also induce anti-inflammatory cytokine expression. It is well known that IL-10 is a relevant inhibitor of the IL-12 secretion [43]. Previous studies demonstrated that mature DCs express specifically natural killer stimulatory factor or IL-12, which plays a key role in triggering T-helper 1 responses [25,44]. In our experiments, we found significantly higher IL-10 and IL-12p40 but not IL-12p35 gene expression levels of DCs cultured in the presence of osteogenically induced JPCs-seeded β-TCP scaffolds. A previous study reported that galectin-1 secreted by MSCs inhibit the function of DCs through downregulation of mitogen-activated protein kinase (MAPK) signaling pathway, whereas high levels of IL-12 and IL-10 were detected in the supernatants [45]. As shown in Figure S2, we also detected high concentrations of galectin-1 and -3 in JPCs at the end of coculture with DCs, suggesting a similar underlaying mechanisms whereby we did not analyze the MAPK pathway. In addition, our results showed increased gene levels of the anti-inflammatory cytokine G-CSF in DCs cultured in the presence of JPCs-seeded β-TCP scaffolds under normal culture conditions (Figure 4). It is reported that G-CSF is considered as an activator of signal transducer and activator of transcription 3 (STAT3) [46]. Activation of the STAT3 pathway was shown to suppress antitumor immunity, specifically by the inhibition of DC differentiation [47,48].

Effects triggered by the addition of osteogenic medium only, in the present study, have to be taken into consideration. We detected an overall suppression of IL-12p35, IL-12p40, both subunits of the IL-12 receptor, and pro-inflammatory cytokine IFN-γ as well as increased chemokine expression levels of MIP-1α/β and the anti-inflammatory cytokine IL-10 (Figure 4). These effects are probably caused by the presence of dexamethasone in the osteogenic medium. Xia and coauthors have proven that dexamethasone promotes the generation of IL-10-producing, immature DCs with little IL-12 production [49]. An additional important aspect is the influence of the DMEM medium in the absence or presence of osteogenic stimuli on the degradation behavior of β-TCP material. This could have an impact on the response of monocultured DCs. In our study, DCs cultured in the presence of β-TCP scaffolds under normal DMEM conditions revealed highest gene expression levels of IL-12 and its receptor subunits as well as highest IFN-γ transcript and protein levels. DCs cultivated in the presence of β-TCP scaffolds under osteogenic conditions showed highly reduced levels of the mentioned cytokines. Lange et al. previously reported that β-TCP particles increased the production of IL-1β, IL-8, TNF-α, and GM-CSF in DCs [50]. The comparison to our results is difficult, while in Lange’s work DCs were generated only by the addition of GM-CSF for 5 days and the β-TCP material was added to the cells in form of a fine particle solution containing particles of a diameter of 0.1–150 μm for 24 h. Further, only RPMI medium was used. On the other hand, Dong et al. demonstrated that BMMSC-seeded β-TCP scaffolds slowed down the degradation of the β-TCP material under osteogenic medium [51]. This result is not unexpected, considering the fact that the scaffold surface is not in direct contact with the medium due to the cell layer covering the surface. However, β-TCP particles phenotypically and functionally stimulated DCs, including upregulated costimulatory molecules CD40, CD80, and CD86, and MHC class II and increased cytokines and chemokines M-CSF, CCL2, and CCL3 [52].

We further investigated cytokine protein production released by DCs. However, a limitation of our study is the lack of validation by other approaches such as ELISA or Western blot. As shown in Figure 5B, G-CSF expression, as an anti-inflammatory immunomodulatory factor, increased significantly by the coculture with JPCs under both culture conditions [53]. In studies examining phenotypical and functional characteristics of human MSCs, G-CSF release in MSC supernatants was almost undetectable [54,55,56]. Similarly, we detected low G-CSF concentrations in JPC supernatants when they were cultured under human platelet lysate (hPL) supplementation and normal unstimulated conditions (Figure S3). Therefore, we can assume that detected G-CSF protein levels in DC supernatants were released by DCs, and not by cocultured JPCs. G-CSF was shown to decrease the expression of the costimulatory molecule CD86 and to inhibit TNF-α and IL-12 production by DCs [57,58]. This mechanism could be also an explanation for IL-12p35 and IL-12p40 repression detected under osteogenic conditions in our study by gene expression analysis. Since IL-12p70 protein levels remained too low, we were not able to detect them by protein analysis. G-CSF as a potent hematopoietic factor can stimulate bone marrow production of granulocytes and hematopoietic stem cells (HSCs), mobilizing them into the bloodstream to replenish lymphoid and myeloid lineages [59,60]. Therefore, G-CSF is used not only to accelerate the recovery of patients with neutropenia but also to increase HSC mobilization into the peripheral blood of the donor before isolation and transplantation of HSCs [59,61]. Additionally, our results from proteome arrays show that in contrast to gene expression analysis, protein levels of the two closely related T cell chemoattractants, MIP-1α and MIP-1β, were significantly reduced under osteogenic culture conditions and, also to a higher extent, by JPCs-seeded constructs (Figure 5B). MIP-1α plays an important role in DCs migration into peripheral tissues [62]. Additionally, MIP-1α/β regulate T lymphocyte trafficking into lymph nodes during immune responses in vivo [63]. It is possible that the suppression of MIP-1α and MIP-1β secretion could be a part of the underlaying mechanism of immunomodulatory JPC functions under osteogenic culture conditions. We found that IFN-γ release showed a similar pattern as MIP-1α/β, and it was significantly suppressed by osteogenic conditions. This effect is probably mainly due to the presence of dexamethasone in the osteogenic medium, since this effect was also described for the human monocytic cell line (THP-1) [64].

## 5. Conclusions

In this study, we demonstrated that JPCs cultured within β-TCP scaffolds suppress monocyte-derived DC maturation in vitro. Our finding is based on detected lower cell densities and mature/immature DC ratios in the presence of JPCs-seeded β-TCP scaffolds, probably attributed to the overall inhibition of pro-inflammatory and induction of the anti-inflammatory cytokines such as IL-10 and G-CSF. Our findings lead to the speculation that JPCs represent a promising stem cell source for bone tissue regeneration. Nevertheless, the underlying mechanism for the suppressive effects of JPCs on DC differentiation should be further investigated.

## Figures and Tables

**Table 1:** Percentages of positive monocytes (control) and dendritic cells (DCs) cocultured with cell-free beta-tricalcium phosphate (β-TCP) or jaw periosteal progenitor cells (JPCs)-seeded β-TCP constructs under normal/osteogenically induced (Co/Ob) conditions for 14 days (β-TCP constructs for 7 days monocultures followed by 7 days cocultures with DCs) for the listed surface antigens.

Surface Markers	Control	Experimental Groups			
		β-TCP/Co	β-TCP/Ob	JPCs + β-TCP/Co	JPCs + β-TCP/Ob
CD14	11.06 ± 6.39	20.85 ± 10.68 ^a^	72.45 ± 3.44 ^a^*	14.27 ± 2.37 ^A^	66.67 ± 1.39 ^A^*
CD83	9.21 ± 4.76	40.85 ± 21.36	38.64 ± 26.19	48.48 ± 9.91	41.38 ± 15.69
CD209	1.03 ± 0.59	14.35 ± 7.32 ^a^	69.50 ± 13.02 ^a^*	28.56 ± 6.19	62.77 ± 13.54 *
HLA-DR	26.74 ± 5.68	91.55 ± 5.86 *	95.83 ± 1.57 *	87.85 ± 6.25 *	93.73 ± 3.59 *

The data of five independent experiments are shown as mean ± SEM. ^a^ Significant differences detected between β-TCP/Co and β-TCP/Ob (p < 0.05) experimental groups. ^A^ Significant differences detected between JPCs + β-TCP/Co and JPCs + β-TCP/Ob (p < 0.05) groups. Superscript asterisks represent statistical significance differences compared to the monocyte control (*p* < 0.05).

## Supplementary Materials

Supplementary materials can be found at https://www.mdpi.com/2218-273X/10/6/887/s1. Figure S1: quantitative gene expression of JPCs seeded within β-TCP constructs cocultured with DCs under Co and Ob conditions for 14 days, Figure S2: protein expression of galectin-1 and galectin-3 in supernatants from JPCs-seeded β-TCP scaffolds before and after coculture with DCs under Co and Ob conditions for 14 days, Figure S3: protein expression of G-CSF in supernatants from JPCs (cultivation in the absence of DCs) cultured under hPL supplementation and Co and Ob conditions for 15 days with or without dexamethasone.
