# Supplementary material for: Jaw Periosteal Cells Seeded in Beta-Tricalcium Phosphate Inhibit Dendritic Cell Maturation
Source: Biomolecules. 2020 Jun 10;10(6):887. doi: 10.3390/biom10060887 (PMC7355649; doi:10.3390/biom10060887)
Supplement: Supplementary file 1 [file biomolecules-10-00887-s001.pdf]

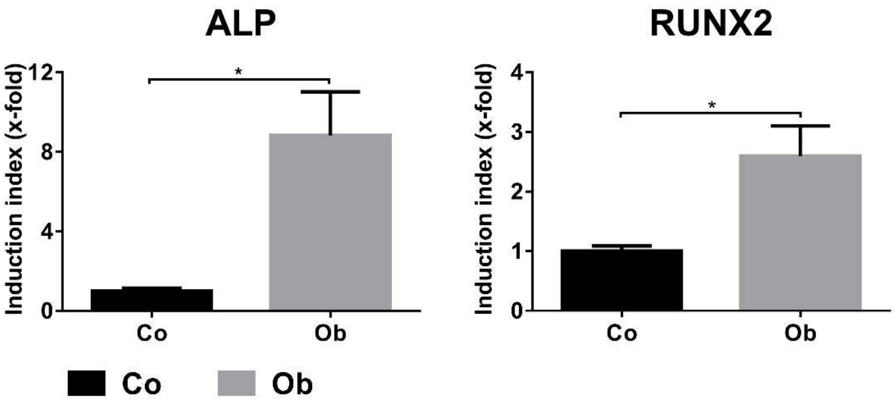

Figure S1. Quantitative gene expression of JPCs seeded within β-TCP constructs and co-cultured with DCs under Co and Ob conditions for 14 days. Tested gene expressions were quantified by the LightCycler System, and ratios of genes in relation to the housekeeping gene glyceraldehyde 3-phosphate dehydrogenase (GAPDH) were calculated. Gene levels in JPCs seeded within β-TCP constructs co-cultured with DCs under normal conditions (Co) was set as 1 (control), and induction indices (x-fold) in relation to the control were calculated. Data were collected from three independent experiments (* $p < 0.05$).

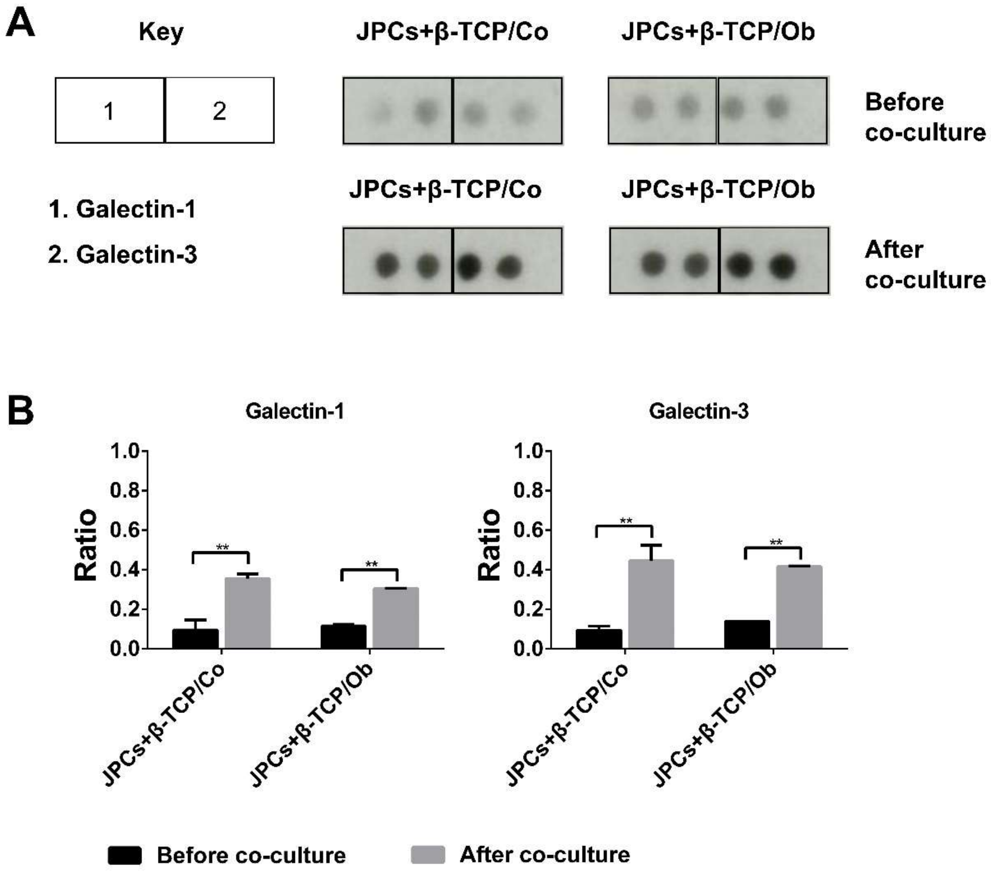

Figure S2. Protein expression of Galectin-1 and Galectin-3 in supernatants from JPCs cultured within β-TCP constructs before and after co-culture with DCs under Co (untreated) and Ob (osteogenic) conditions for 14 days (7 days monocultures followed by 7 days co-culture). (A) Representative images of proteome profiler arrays (human soluble receptor array kit); (B) Quantification analysis of the protein expression by ImageJ software. Data were collected from three independent experiments (**$p < 0.01$).

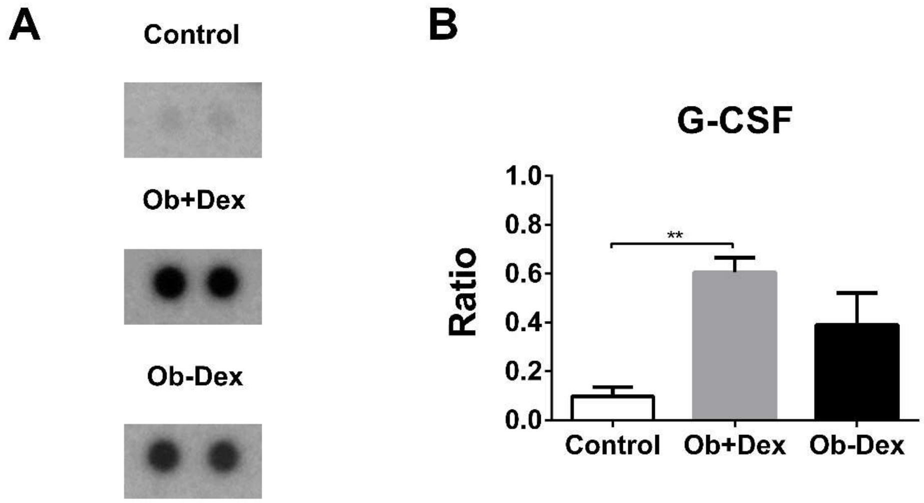

Figure S3. Protein expression of G-CSF in supernatants from JPCs (cultivation in the absence of DCs) cultured under human platelet lysate (hPL) supplementation and Co (untreated) and Ob (osteogenic) conditions for 15 days with or without dexamethasone. (A) Representative images of proteome profiler arrays (human cytokine array kit); (B) Quantification analysis of G-CSF protein expression by ImageJ software. Data were collected from three independent experiments (**$p < 0.01$).
